# Application of choosing by advantages to determine the optimal site for solar power plants

**DOI:** 10.1038/s41598-022-08193-1

**Published:** 2022-03-08

**Authors:** Hui Hwang Goh, Chunyu Li, Dongdong Zhang, Wei Dai, Chee Shen Lim, Tonni Agustiono Kurniawan, Kai Chen Goh

**Affiliations:** 1 School of Electrical Engineering, Guangxi University, Nanning, Guangxi China; 2 Department of Electrical and Electronic Engineering, School of Advanced Technology, Xi’an Jiaotong-Liverpool University, Suzhou, 215123 China; 3 College of Environment and Ecology, Xiamen University, Fujian, 361102 China; 4 Department of Technology Management, Faculty of Construction Management and Business, University Tun Hussein Onn Malaysia, 86400 Parit Raja, Johor Malaysia

**Keywords:** Environmental impact, Energy and society, Solar energy

## Abstract

Solar energy is a critical component of the energy development strategy. The site selection for solar power plants has a significant impact on the cost of energy production. A favorable situation would result in significant cost savings and increased electricity generation efficiency. California is located in the southwest region of the United States of America and is blessed with an abundance of sunlight. In recent years, the state's economy and population have expanded quickly, resulting in an increased need for power. This study examines the south of California as a possibly well-suited site for the constructing large solar power plants to meet the local electricity needs. To begin, this article imposed some limits on the selection of three potential sites for constructing solar power plants (S1, S2, and S3). Then, a systematic approach for solar power plant site selection was presented, focusing on five major factors (economic, technological, social, geographical, and environmental). This is the first time that the choosing by advantages (CBA) method has been used to determine the optimal sites for solar power plant construction, with the possible sites ranked as S2 > S1 > S3. The results were then compared with traditional methods such as the multi-criteria decision-making method. The findings of this study suggest that the CBA method not only streamlines the solar power plant site selection process but also closely aligns with the objectives and desires of the investors.

## Introduction

Historically, nonrenewable energy sources such as fossil fuels have been heavily relied upon to meet the energy requirements. However, its usage results in significant harmful gas emissions, which has a detrimental effect on the environment and the long-term growth of society^1^. In contrast, solar energy has the advantages of clean and low carbon emissions, which make it widely used in our life^2^. In recent years, solar energy is flourishing in different populated regions of the world to meet our energy needs and to preserve the environment.

Solar power generation is the most common way to use solar energy because of its ease of maintenance and low environmental impact. Solar power generation is predicted to significantly develop in the near future, particularly in industrial areas^3^. In the European Union (EU), solar energy is being used on a large scale to reduce the total carbon dioxide emissions^4^. According to the California Energy Commission report, by implementing solar power in the energy grid, California would roughly triple its existing electrical grid capacity and maintain a record rate of renewable energy capacity expansion over the next 25 years to achieve the state's economy-wide climate goals^5^. In this context, increasingly more solar power plants will be installed in the next decade.

However, increasing the number of solar power plants will be challenging. The lifespan of a solar power plant is roughly 25–30 years^6^. Thus, extending the lifespan of solar power plants and overcoming environmental hurdles posed by decommissioned plants at the end of their lifespan are popular topics of discussion. According to Domínguez, as more solar power plants are built, the amount of photovoltaic (PV) waste produced will dramatically increase^7^. Based on this, Farrell et al. reviewed and analyzed the recycling approaches of PV waste and assessed the potential energy value of waste PV modules to realize circular economy (CE)^8,9^. In the past few years, enormous progress has been made in the application and implementation of CE worldwide. The European Commission formulated a CE plan for the sustainable development of the EU in 2015^10^. Many policymakers and stakeholders are seeking to apply CE to various fields, with the solar power industry leading the way. Solar power plant construction is the basis of realizing solar energy CE. This enhances coherence among environment, economy, and society, which creates a sustainable business environment for investors.

To maximize the CE benefits of the solar power industry, the optimal site must be found for the construction of solar power plants, which requires a balance of economy, society, environment, and climate, and is regarded as a multi-criteria decision-making (MCDM) problem^11^. The existing literature mostly considers economic, environmental, and technological factors, but social factors, such as population density, are rarely mentioned^12–14^. With the rapid increase in the world population, factors related to social influence and human behavior are of great concern to decision-makers. Therefore, a comprehensive and connotative site selection model needs to be put forward to meet the site selection requirements. Herein, a new site selection model is proposed based on a comprehensive research background, considering economy, technology, society, geography, and environment.

Nevertheless, the realization of CE is affected by the investment decisions made by stakeholders considering the high costs of solar power plant construction. For investors, projects will not be selected that have low investment returns^15,16^. As a result, when faced with high-cost investments, stakeholders need to analyze the costs separately to make the risks of the projects transparent^17^. The investment cost of solar power plants is 4739 $/kW, while the investment cost of concentrating solar power plants is 5213–6672 $/kW in the United States of America^18^. The construction cost of the Crescent Dunes Solar Energy Project was $1 billion in 2015^19^. Therefore, due to the high construction costs, the investment cost in the solar power plant construction needs to be considered and analyzed to make these projects profitable for the investors. Existing studies treat cost factor by comparing its importance with other factors, which do not highlight the importance of cost and makes cost insensitive to the impact of site selection^20,21^. To fill this research gap, this paper considers cost as an independent factor in the process of solar power plant site selection to reflect the value of cost and to maximize investors’ return on investment.

In order to provide a comprehensive research background and reflect the value of cost, a new choosing by advantages (CBA) method is applied in this paper. The main contributions of this paper are as follows:

1. This paper created a comprehensive and methodical scheme for solar power plant site selection, which includes five basic factors and corresponding sub-factors: economy, technology, society, geography, and environment. Then, considering the high investment cost of solar power plant construction, this paper separates the cost from other factors to maximize investors' return on investment. The scheme is applied to support the site selection of solar power plants in California.
2. The CBA method is firstly used in the site selection for large solar power plants, and it provides a new solution for adequate decision-making.

This paper primarily aims to propose a valuable and meaningful scheme of solar power plant site selection to provide technical support for the realization of solar energy CE. The remainder of the study is divided into the following sections: “Literature review” section provides a brief review of the MCDM method and its application to the optimal site selection of solar power plants. “Methodology” section examines the criteria, parameters, and model for the solar power plant site; it also includes specifics on the CBA method. In “Results and discussion” section, the results are discussed, and the CBA sensitivity analysis is conducted. Finally, “Conclusion” section interprets the paper's conclusions.

## Literature review

In this section, the existing research on the current MCDM methods and their application to the optimal site selection of solar power plants are briefly reviewed. MCDM is a well-known decision-making approach in operations research that encompasses a variety of techniques. Tirkolaee and his team have used MCDM to solve a series of decision-making problems, including supplier selection in the healthcare industry, enterprise business plan decision-making, and the optimal allocation of energy^22–24^. In recent years, the decision-making problems have gradually developed into complex MCDM problems, which are often accompanied by the subjectivity of decision-makers and the uncertainty of information.

Based on this, the fuzzy theory and concept have been developed to meet the decision-making requirement. Ali et al. proposed a complex interval-valued Pythagorean fuzzy set for green supplier chain management selection^25^. Sahu et al. proposed a method based on picture fuzzy set and rough set to solve the decision-making problem^26^. However, these methods cannot deal with soft multiset scenarios. To overcome this challenge, the concept of soft multiset and soft multiset topology are extended by Riaz to solve the MCDM problems^27^.

Progressively more MCDM methods have been developed by combining with the fuzzy concept and theory. Mishra et al. combined the technique for order preference by similarity ideal solution (TOPSIS) method with intuitionistic fuzzy weighted measures to solve the decision-making problem of the investment policy choice^28^. TOPSIS is an MCDM method based on the distance between positive and negative ideal solutions (PIS and NIS, respectively). Rani et al. extended fuzzy TOPSIS with the new divergence measures to select renewable energy sources^29^. At present, TOPSIS has proven to have good applicability in various fields, especially in site selection^30^.

The measurement alternatives and ranking according to compromise solution (MARCOS) method was developed by Stevic et al. based on the idea of TOPSIS^31^. Uluta et al. further extended MARCOS with correlation coefficient and standard deviation (CCSD) and indifference threshold-based attribute ratio analysis (ITARA) methods to the logistics system^32^. However, the main limitation of this method is that it is difficult to express the evaluation criteria correctly through explicit numerical values. Therefore, Brkovic et al.^33^ presented an integrated full consistency method–MARCOS model, and Celik et al.^34^ integrated the best–worst method (BWM), MARCOS, and interval type-2 fuzzy sets to avoid this limitation. From the perspective of application, the MARCOS method’s applicability in the field of site selection has not yet been proven.

Multi-attributive border approximation area comparison (MABAC) is an area boundary approximation method, and Pamucar et al. extended different MABAC methods to solve different decision problem^35,36^. Wang et al. developed an improved MABAC method based on the q-rung orthopair fuzzy set (Q-ROFS) environment. However, due to the limited practical use of Q-ROFS and MABAC, this combination method may not be appropriate for use in real-life problems^37^. Similar to the TOPSIS, MARCOS, and MABAC methods, the multi-attribute ideal–real comparative analysis (MAIRCA) and Vlsekriterijumska Optimizacija I Kompromisno Resenje (VIKOR) methods were combined with fuzzy concept, such as fuzzy analytic hierarchy process (FAHP)-VIKOR^38^ and FAHP-MAIRCA methods^39^.

Different from TOPSIS, MARCOS, MABAC, MAIRCA, and VIKOR, preference ranking organization method for enrichment evaluation (PROMETHEE) is an outranking method. Researchers extended the PROMETHEE method to different scenarios. A fuzzy PROMETHEE method combined with trapezoidal fuzzy interval numbers has been applied to the automobile industry^40^. The PROMETHEE method with intuitionistic fuzzy soft sets has been extended to solve the decision-making problems with intuitionistic fuzzy information. The PROMETHEE method has great advantages when decisions to be made by experts are influenced by their respective areas of expertise, so it has been widely used for site selection^41,42^.

The main form of the current MCDM methods is in combination with fuzzy concept, weight determination methods, and ranking methods. This proves that the current MCDM methods are mature and can be effectively applied to decision-making involving a large number of fuzzy and uncertain factors and information, such as the site selection for solar power plants. TOPSIS^43^, PROMETHEE^44^, and VIKOR^45^ have been proven to have good performance in the field of solar power plant site selection. However, in the application of TOPSIS, the factors of solar power plant site selection are not fully considered such as geographical disasters, population density, and visual impact^43^. In PROMETHEE^44^, payback period, population density, and policies are not taken into account^45^. Factors such as geographical disasters and policies are also not mentioned in VIKOR. In addition, the cost is not considered as a single component but compared with other factors in the TOPSIS, PROMETHEE, and VIKOR methods, which hides the true value of cost and reduces its influence on the decision-making results.

To overcome the challenges of the TOPSIS, PROMETHEE, and VIKOR methods in solar power plant site selection, this paper proposes a more comprehensive and meaningful scheme that incorporates CBA method and a solar power plant model involving economic, technological, geographical, environmental, and social factors. This scheme can also maximize the interests of investors and the CE of solar power projects based on the CBA method. The CBA method is a lean decision-making method built by Suhr in 1999 that supports sound decision-making using alternative advantage comparisons^46^. It can solve the MCDM problems and separate cost from other factors in the process of decision-making to fully ensure the real value of cost. The CBA method has been successfully applied in the architecture, engineering, and construction industry and has proven to be better than other traditional approaches^47–49^. The advantages of the CBA method are as follows^28^: (1) It provides a more transparent environment for the decision-makers. (2) It can be closely related to the context of the project, reducing the time for decision-makers to reach consensus. (3) Cost factors are considered separately to ensure its importance on decision-making results. Therefore, the CBA method is adopted for the optimal site selection for solar power plants in this study. Table 1 summarizes some advantages and limitations of the abovementioned approaches.

**Table 1:** The advantages and limitations of several different decision-making methods^31,48,50,51^.

Category	Methods	Advantages	Limitations	
Distance methods	MARCOS	It considered the nonideal solution and ideal solution before the formation of the initial matrix	Its applicability in most areas is yet to be demonstrated	These methods compared the importance of the factors to determine their weights, ignoring the advantages of those factors
	TOPSIS	It allows to interpret the absolute evaluation of certain site alternatives	Its Euclidean distance does focus on the correlation of the attributes	
	MAIRCA	It uses a simple algorithm	Its applicability to the optimal site selection of solar power plants has not been proven	
	VIKOR	It is based on the principle of compromise programming for multimode multiplexing systems	It needs initial weights	
	MABAC	The formula is very simple	The distance measurement is inadequate	
Outranking methods	PROMETHEE	It does not require the criteria to be proportionate	It does not provide a clear framework for assigning the weights	
Lean thinking method	CBA	It considers the importance of factors' advantages rather than the importance of the factors themselves. Cost is considered as a separate factor	It requires a deep understanding of alternatives	

## Methodology

### Establish the criteria and factors

Following a comprehensive review of the relevant literature and consultation with industry experts, this paper suggests 16 essential site selection factors. However, at some point throughout the site selection process, the characteristics of factors may have an effect on the output’s accuracy. To ideally solve this problem, the factors considered in this study can be classified as positive or negative, based on whether or not they contribute positively solar power plant production enhancement, respectively. Visual impact, solar irradiation potential, land type, geological disaster, policies, public attitude, and local development planning are considered beneficial criteria; in contrast, payback period, investment cost, rainfall, temperature, humidity, distance to roads, distance to substations, and population density are considered detrimental criteria. This treatment would advocate for simplifying the MCDM model and outlining the CBA model’s decision-making rules. The justification and explanation for the selection of each factor is discussed in greater detail below:

- *Visual impact* The construction of solar power plants would have an effect on the daily life of animals and humans^52^. To maintain the long-term viability of the ecosystem, the visual impact of solar power plants must be considered during the design stage.
- *Solar irradiation potential* This is clearly the key indicator determining whether solar power plants can be built at a particular site. Solar power plant’s ability to produce energy and save money is directly impacted by the amount of available solar energy. With higher amount of solar radiation being available, more electricity can be generated, making the electricity grid more efficient^53^.
- *Land type* In some places, the land type and availability might be a critical factor in determining the site for solar power plant construction. Numerous countries have regulations regarding the types of land that can be used for solar power projects. Generally, it is preferable to employ construction land rather than agricultural land, as this would contravene the principle of sustainable growth.
- *Geological disaster* This is a critical geographical factor in the development of solar power plants. If an area is prone to geological disasters, such as tsunamis and earthquakes, investors will encounter significant risks, and there is no value in installing solar power plants in such areas.
- *Policy* It is critical to consider local policies for site selection. Solar energy generation is expensive due to technical constraints. When a country or municipal government reduces taxes while increasing energy prices, the investment rate increases, relieving the financial pressure on investors.
- *Social benefit* Solar power plants are built to meet the interests of investors while also positively contributing to society. They will assist in promoting local businesses and creating jobs, thereby impacting local education and culture^54^.
- *Public attitude* The development of large solar power plants is a massive and time-consuming endeavor. They often have detrimental effects on nearby inhabitants in terms of noise for example. It is necessary to perform extensive research to ascertain whether the local populace supports solar power plant construction.
- *Local development planning* This serves as the foundation for the investment and commercial decision-making. If the local economy and social system have remained stagnant and saturated, the viability and hazards of investing in solar power plants must be evaluated.
- *Payback period* This is a critical factor to examine when determining whether a project is worth investing in, and it is also a benchmark for decision-makers when determining a project’s profitability. When selecting a site for solar power plants, a project with a lengthy payback period is inappropriate and should not be prioritized.
- *Investment cost* This is a critical factor when undertaking any project. It weighs the project’s expenses and benefits, and its appropriate consideration would lead to a cost-effective and dependable solution. The investment cost primarily encompasses the costs for land acquisition in this paper.
- *Rainfall* Rainfall may damage solar panels and other construction equipment, reducing their lifespan. Solar power plants should be constructed with extreme caution in places prone to excessive precipitation.
- *Temperature* Temperature can affect the longevity of solar power plants. Increased temperature can reduce the efficiency of solar energy conversion devices, resulting in decreased output^55^. When the average temperature is maintained at a steady and acceptable level, solar power plants can operate at maximum capacity.
- *Humidity* Increased humidity results in less solar radiation, lowering the performance of solar energy conversion, increasing the cost of power generation^56^.
- *Distance to roads/substations* The technical strategy must account for the distance between solar power plants and roads and substations. Solar power plants built near transformer substations will help reduce equipment transportation costs and enable easier construction of new infrastructure.
- *Population density* This illustrates how metropolitan systems evolve. The population distribution and density are also critical variables in the solar power plant site selection process.

All of the abovementioned factors were determined with the assistance of experts and relevant institutions from around the world to bolster the viability of the site selection system and data dependability. Experts include local governments, government agencies, consultants, renewable energy specialists, project managers, quantity surveyors, engineers, architects, scientists, and stakeholders. Their knowledge and abilities ensure the logic and dependability of the system.

### The procedure for the optimal site selection for a solar power plant

This research evaluates the economic, technological, environmental, geographical, and social factors of the study region, as well as the potential for solar power generation growth, to maximize the benefits from a solar power plant. A precise approach for the site selection of solar power plants has been developed.

**Figure 1:**
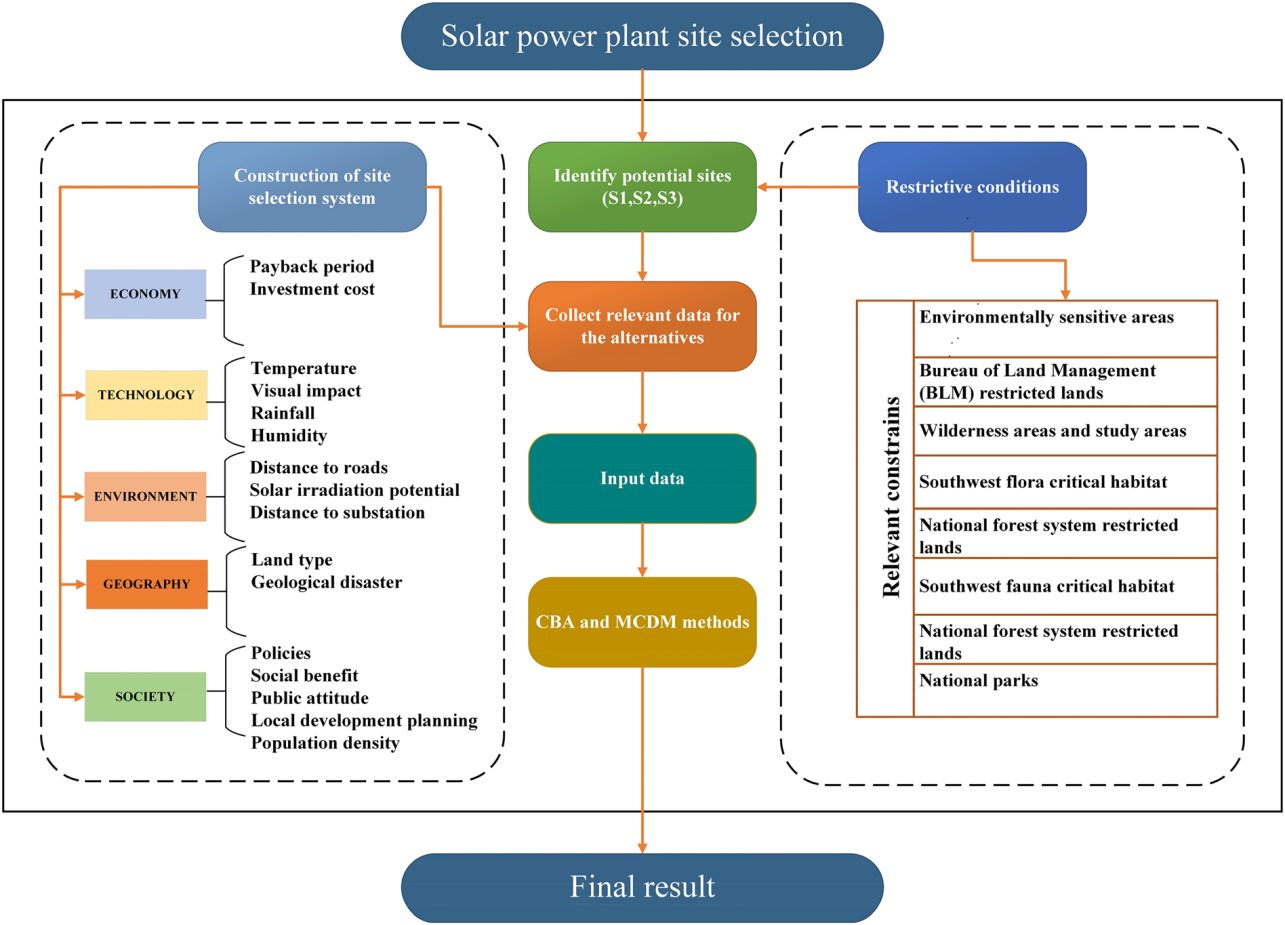
Solar power plant site selection framework.

Figure 1 illustrates the process of choosing a site for a solar power plant construction. The specific steps are described below:

Step 1: Create a site selection model based on the 16 factors and suggest some constraints to help define possible site alternatives (S1, S2, and S3).
Step 2: Collect and evaluate relevant data for each site alternative in accordance with the site selection method.
Step 3: Determine the optimal site using the CBA model.

This approach would improve the precision and objectivity of the site selection process’s outcome. It must be noted that due to the low slope angle of the land in the study field, the slope and orientation of the land are not included in this research.

### Study area and data collection

**Figure 2:**
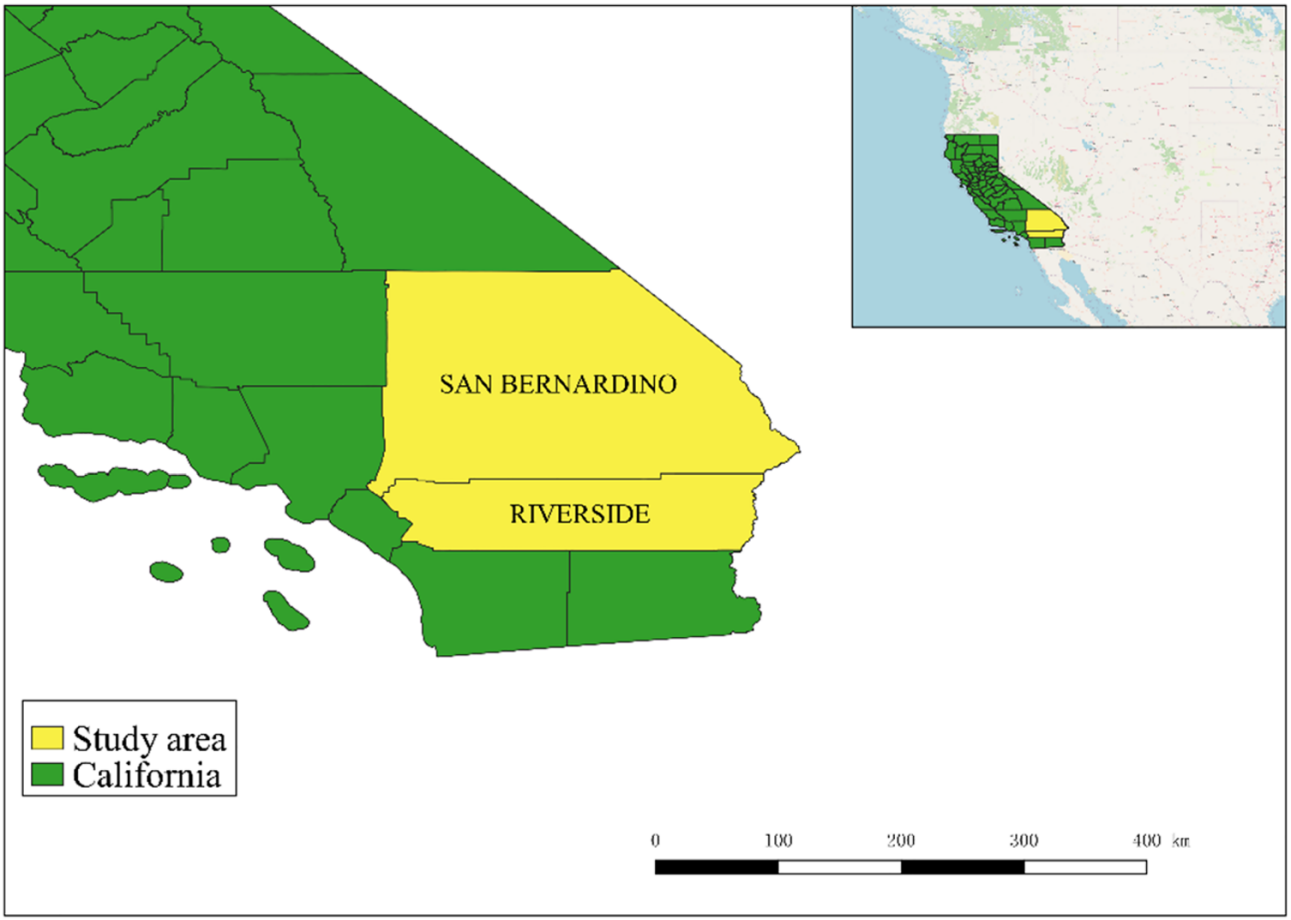
Map of the study area (this image was created by QGIS software V3.14.16-Pi, URL link: https://www.qgis.org/en/site/)^57^.

This study focused on the southern California counties of San Bernardino and Riverside (Fig. 2), which are mostly deserts, sparsely populated, and bountiful in solar energy. As a result, the majority of California’s solar projects are located in those two counties to supply electricity to western California’s metropolitan clusters. To begin, the factors indicated in Fig. 1 were used to select three suitable solar project sites (S1, S2, and S3). Subsequently, specifics about possible sites are provided. Prior to analyzing the site alternatives, this study’s data were collected, which are show in Table 2. All data and statistics are derived from a variety of sources, including the National Renewable Energy Laboratory, the Weather Atlas website, and the Bureau of Land Management.

**Table 2:** CBA tabular method.

Factors	Site	Attribute	Advantages		
**1. Payback period** *Criteria: The shorter the period, the better*	S1	18.75 years	It is the second shortest payback period		78
	S2	***18.71 years***	***It is the shortest payback period***		80
	S3	19.51 years	–		–
**2. Temperature** *Criteria: The lower temperature, the better*	S1	***33.40 °C***	***It is the most suitable temperature***		55
	S2	34.00 °C	It is the more suitable temperature		52
	S3	40.70 °C	–		–
**3. Visual impact** *Criteria: The less vegetation or wildlife on the site, the better*	S1	***It is a desert valley with little flora***	***Much better for solar power plant construction***		10
	S2	It is a place with a diverse vegetation	–		–
	S3	***It is a valley with little tall vegetation***	***Much better for solar power plant construction***		10
**4.Rainfall** *Criteria: The less the rainfall, the better*	S1	10.20 mm	–		–
	S2	9.75 mm	Less annual rainfall		5
	S3	***6.10 mm***	***Minimum annual rainfall***		20
**5. Humidity** *Criteria: The less the humidity, the better*	S1	39.60%	–		–
	S2	37.80%	The second lowest value of the three sites		5
	S3	***27.40%***	***The lowest value of the three sites***		15
**6. Distance to substations** *Criteria: The shorter the distance, the better*	S1	***11.57 km***	***It is closest to the substation***		60
	S2	15.53 km	It is closer to the substation		45
	S3	16.39 km	–		–
**7. Distance to roads** *Criteria: The shorter the distance, the better*	S1	***7.13 km***	***The site is the closest to the road, significantly lowering the equipment's transportation costs***		50
	S2	12.46 km	–		–
	S3	7.17 km	The location is relatively close to the road, which reduces the equipment’s transportation cost marginally		30
**8. Solar irradiation potential** *Criteria: The higher the intensity of solar radiation, the better*	S1	***5.95 kW/m***^***2***^***/day***	***It possesses the world's most plentiful solar energy resources***		100
	S2	5.78 kW/m^2^/day	There is sufficient solar energy available to build a solar power plant		90
	S3	5.71 kW/m^2^/day	–		–
**9. Land type** *Criteria: Deserts were favored, followed by valleys*	S1	The area is classified as a high desert and is zoned for construction	Solar power plants are more suited for construction land		20
	S2	The land is primarily used to build tourist amenities	–		–
	S3	***This area is primarily made up of building land and desert wasteland***	***Desert wasteland has a cheap cost of land***		30
**10. Geological disaster** *Criteria: Preferably no geological disasters*	S1	There are may has significant natural disasters and looming floods	–		–
	S2	There have been no significant natural catastrophes	The solar facility's life will be extended, and the project's development will be safer		65
	S3	***The site is located on a fault line, which means that mild earthquakes are possible***	***Minor tremors have little effect on the stability of solar-powered equipment***		60
**11. Policies** *Criteria: The more positive the impact on the construction of solar power plants, the better*	S1	There is little demand for electricity in this area	–		–
	S2	This area's electricity is mostly used to support local tourism growth	This location has a certain demand for electricity		70
	S3	***This area will serve as a power transmission link between California and Arizona***	***There are significant benefits of promoting solar power plant development***		85
**12. Social benefit** *Criteria: The more benefits to society, the better*	S1	***Building the solar power plant can boost economic development in the area***	***Promotes local economic, social, and cultural development***		70
	S2	Building the solar power plant will help meet the tourism industry's electricity needs	Mainly promotes local tourism		65
	S3	Constructing a solar power plant can result in an increase in employment	–		–
**13. Public attitude** *Criteria: Prefer a positive attitude*	S1	Most individuals are opposed to solar power plant construction	–		–
	S2	The majority of people support the construction of solar power plants	It will significantly mitigate the impact of human variables		35
	S3	***Almost all of people advocate for constructing solar power plants***	***It will eliminate all human-caused influences***		45
**14. Local development planning** *Criteria: The more site alternatives that promote local development, the better*	S1	Solar power plants may not be included in the development plan	–		–
	S2	Solar power plants are only a modest portion of the development plan	Slightly promotes local development		65
	S3	***Solar power plants are a critical component of the development plan***	***Greatly promotes local development***		75
**15.Population density** *Criteria: The smaller the population density, the better*	S1	640.96/km^2^	–		–
	S2	***198.15/km***^***2***^	***There is plenty of available land space***		40
	S3	285.87/km^2^	There is a small amount of available land space		30
Cost (million dollar):		S1: 3.96	S2: 3.82	S3: 2.98	
Total IofAs divided by 100:		S1: 4.43	S2: 6.17	S3: 4.00	
I/C (IofAs/cost)		S1: 1.119	S2: 1.615	S3: 1.342	

Significant values are in bold.

### Choosing by advantages method

**Figure 3:**
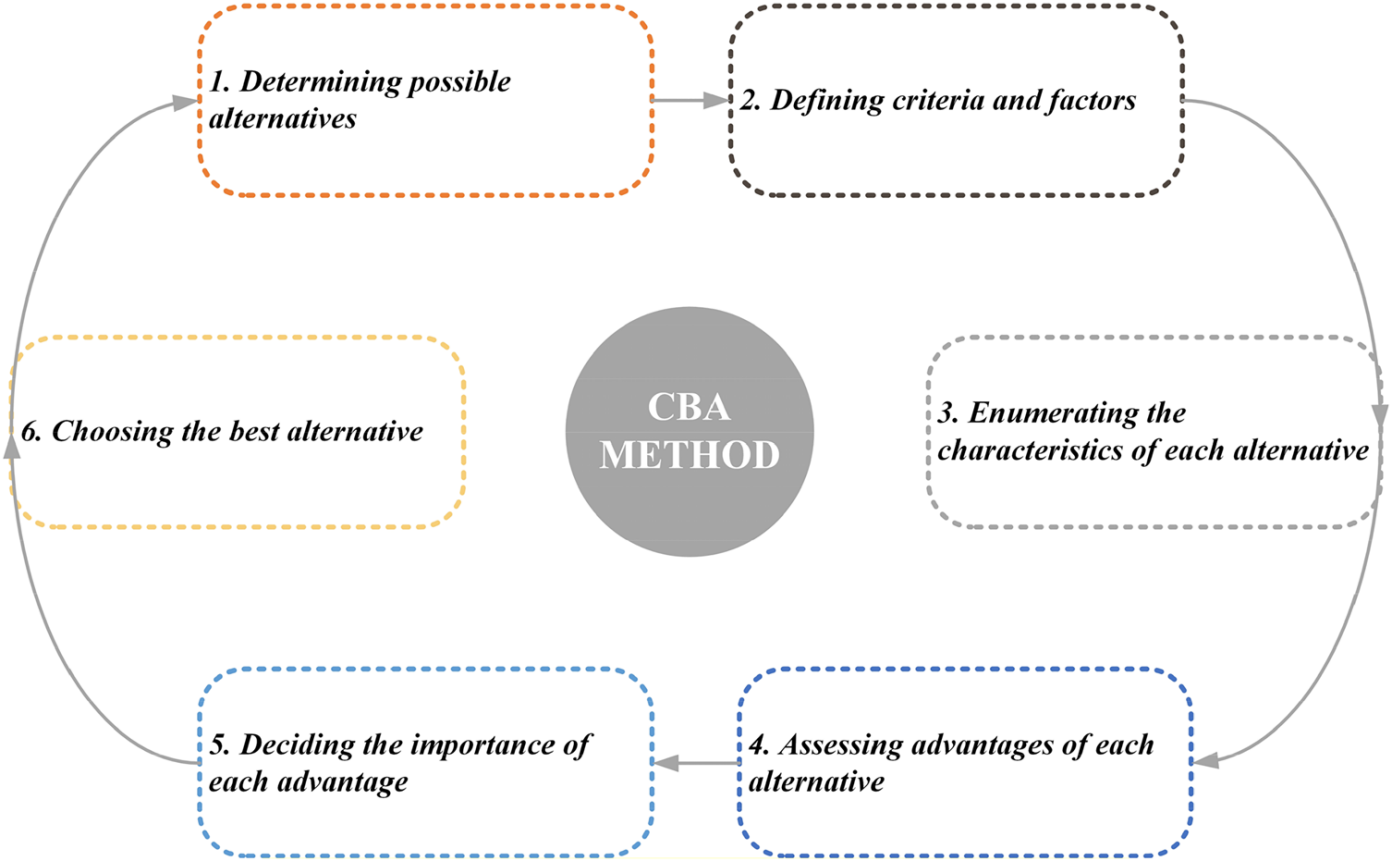
Steps in the CBA method^58^.

CBA’s tabular approach is utilized for solar power plant site selection. As illustrated in Fig. 3, the tabular CBA method comprises of six steps^58^:

1. Determining possible site alternatives. In this study, three possible site alternatives (S1, S2, and S3) are ultimately produced by imposing some constraints on the investigation. These are the site alternatives that are used to conduct the evaluation.
2. Defining criteria and factors. “Literature review” section discusses the criteria and factors that influence the site selection for solar power plants. It is worth emphasizing that the majority of the criteria and factors are quantitative, which makes the CBA method’s decision-making outputs objective and reliable.
3. Enumerating the characteristics of each site alternative. This process involves the experts and stakeholders developing choice rules for each criterion and factor, as well as summarizing the qualities of each site alternative.
4. Assessing advantages of each site alternative. This step requires the stakeholders to evaluate the merits of each site alternative based on the specified criteria and factors, which should be a straightforward undertaking.
5. Deciding the importance of each advantage. The decision-makers should prioritize each advantage. Participants used a scale ranging from 1 to 100 to assign varying degrees of importance. To begin, the “most critical advantage” should receive a score of 100. The following goal is to utilize the “most critical advantage” as a baseline against which the remaining advantages can be compared. The final stage is to determine each site alternative’s total importance of advantages (IofAs).
6. Choosing the best site alternative. The cost of each site alternative is calculated to obtain the cost–IofAs curve. The site alternative that gives the most value for money should be chosen by the stakeholders and decision-makers.

## Results and discussion

### Results of choosing by advantages

**Figure 4:**
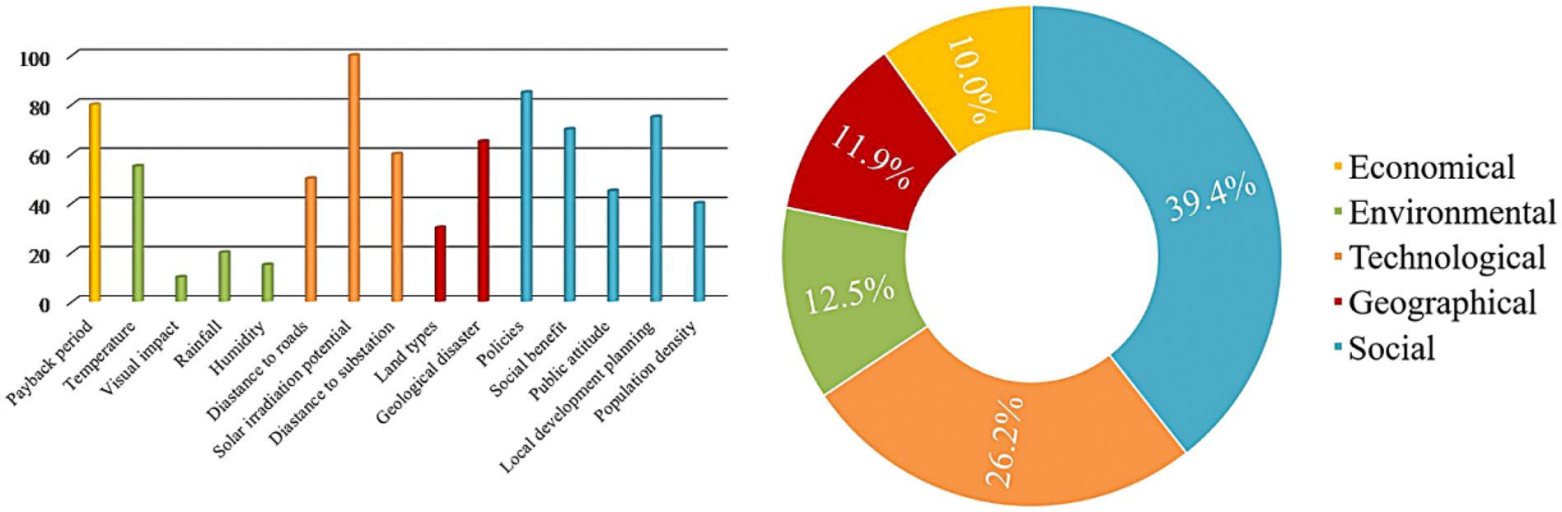
The score distribution of each factor’s advantage.

In contrast to the standard MCDM method, the CBA method places a premium on the relative advantages of the factors rather than their relative importance. To confirm the accuracy of the data and the method's viability, experts from around the world were enlisted to define the criteria and weigh the relative merits of each choice. As a consequence, 15 decision-making factors and criteria (left column of Table 2) were found, with the exception of investment cost. Figure 4 illustrates the score assigned by the experts to each factor's advantage. Clearly, professionals prefer solar irradiation potential, which has a maximum score of 100 and corresponds to the basic understanding of solar energy generation. Additionally, the overall score for technical and social variables is high, showing that decision-makers place a premium on the benefits of these two factors when selecting a solar power plant site.

**Figure 5:**
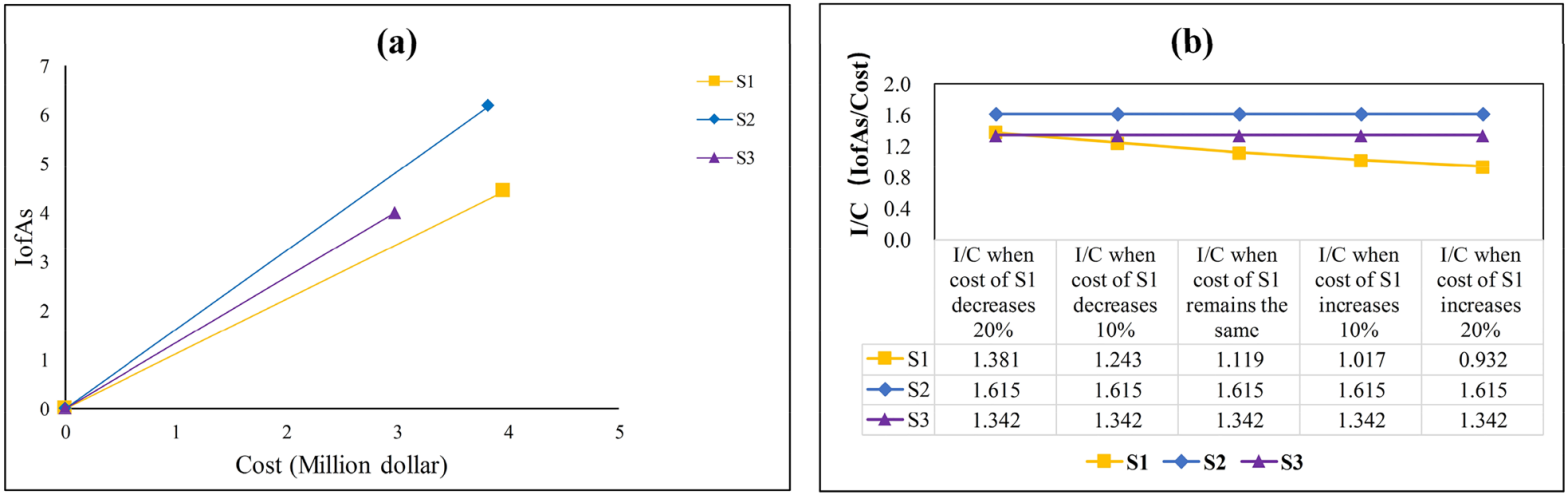
(**a**) The ranking result of CBA method; (**b**) The final result of CBA method when the costs in S1 change.

Table 2 demonstrates how the CBA method can be used to organize data in a way that makes selecting the ideal solar power plant site easier for experts and stakeholders. It can be seen that the relevant factors of each site alternative for a specific project are described in detail in the CBA model, which is helpful for decision-makers to reach a consensus quickly. To facilitate the analysis of the results, the IofAs values in this study were divided by 100. It can be seen that S2 has the highest total score of 6.17, while S1 and S3 scored 4.43 and 4.00, respectively. Figure 5a shows how the CBA model makes decisions based on the cost and IofAs of each site alternative. Clearly, S2 had the second lowest cost and the highest IofAs value when compared to S1 and S3. S1 and S3 have similar IofAs values; however, S3 is substantially less expensive. In conclusion, S2 is the optimal site for solar power plant construction using the CBA method due to its higher cost performance, and the final ranking is S2 > S3 > S1. It can be seen that the impact of cost on the results is fully demonstrated by the CBA method.

Additionally, decision-makers can use the CBA method for decision-making based on their own needs in response to cost changes. Figure 5b illustrates the decision-making outcome when the costs for S1 vary proportionately in this study. Clearly, as the cost of S1 is reduced, its cost performance improves. When the cost of S1 is lowered by approximately 20%, its cost performance index (I/C; the value of IofAs divided by the cost) is greater than that of S3. This signifies that S1 outperforms S3 in terms of the cost performance, and the findings of the CBA method will be changed to S2 > S1 > S3. As can be seen, the CBA method provides a flexible cost analysis, which gives decision-makers more choice.

### Comparison study

To verify the advantages of the CBA method, this study used the TOPSIS and PROMETHEE methods for comparison. Among the distance methods mentioned in “Literature review” section, TOPSIS is one of the most mature methods applied to solar power plant site selection, which ensures the applicability of the method and the reliability of the results^59^. PROMETHEE is an outranking method, and its applicability to solar power plant site selection has been proven^42^. Therefore, by comparing the CBA method proposed in this paper with TOPSIS and PROMEHTEE can not only ensure the reliability and representativeness of comparison but also clearly show the changes in the results for the different methods.

To avoid the unrepresentativeness of the data, experts and stakeholders in the solar industry were asked to determine the importance of the factors used in the TOPSIS and PROMETHEE methods. The obtained data will be converted into triangular fuzzy numbers according to the rules listed in Table 3 and inputted as parameters into the FAHP model to obtain the final weight, as shown in Table 4. Their knowledge and abilities ensure the availability and objectivity of the data. According to Table 5, the ranking result based on closeness coefficients obtained from the standard TOPSIS method is S2 = 0.564 > S1 = 0.488 > S3 = 0.473. S1 is determined to be the most appropriate site for the solar power plant construction due to its high closeness coefficient value. Similarly, S3, with the lowest closeness coefficient value, was identified as the least preferred solution due to its proximity to PIS and to NIS. The final result of the PROMETHEE method is S2 = 0.045 > S1 = 0.029 > S3 =  − 0.073. This demonstrates that the classic MCDM method has the same decision-making performance.

**Table 3:** Rules of transforming regular numbers into triangular fuzzy numbers^60^.

Linguistic scale for importance	Scale value	Triangular fuzzy numbers (TFN)
Absolutely more important (AMI)	9	(7,9,9)
Very strongly more important (VSMI)	7	(5.7.9)
Strongly more important (SMI)	5	(3,5,7)
Weakly more important (WMI)	3	(1,3,5)
Equally important (EI)	1	(0,0,1)
Weakly low important (WLI)	1/3	(1,1/3,1/5)
Strongly low important (SLI)	1/5	(1/3,1/5,1/7)
Very strongly low important (VSLI)	1/7	(1/5,1/7.1/9)
Absolutely low important (ALI)	1/9	(1/7,1/9,1/9)

**Table 4:** The weight of each criterion.

Criteria	Weight (${W}_{j}$)
Payback period	0.061
Investment cost	0.229
Temperature	0.028
Visual impact	0.021
Rainfall	0.010
Humidity	0.010
Distance to roads	0.083
Solar irradiation potential	0.144
Distance to substations	0.093
Land type	0.061
Geological disaster	0.075
Policies	0.062
Social benefit	0.045
Public attitude	0.030
Local development planning	0.028
Population density	0.020

**Table 5:** The final ranking of the three methods.

Site alternatives	Conventional MCDM methods				CBA method			
	TOPSIS		PROMETHEE					
	Closeness coefficients	Ranking	Net outranking flow	Ranking	IofAs	Cost (Million dollar)	I/C (IofAs divided by Cost)	Ranking
S1	0.488	2	0.029	2	4.43	3.96	1.119	3
S2	0.564	1	0.045	1	6.42	3.82	1.615	1
S3	0.473	3	− 0.073	3	4.00	2.98	1.342	2

Clearly, this result is not the same as that obtained using the proposed CBA method (S2 > S3 > S1). The fundamental reason for this is that the investment cost was factored into the TOPSIS and PROMETHEE model evaluations at the beginning, and as a result, S3 scored better than S1 due to its superior performance of other factors. In other words, the disadvantage of S3’s investment cost is outweighed by its other benefits. As a result, when traditional MCDM methods are used for decision-making, the investment cost is weighed against other factors. Unlike the typical MCDM model, the CBA model incorporates the predicted investment cost of each choice as an independent factor to constrain the result. That is, despite the fact that S1 performed brilliantly in this study and achieved a high score in a multitude of areas, due to the high estimated investment cost, investors and decision-makers will not select it.

**Figure 6:**
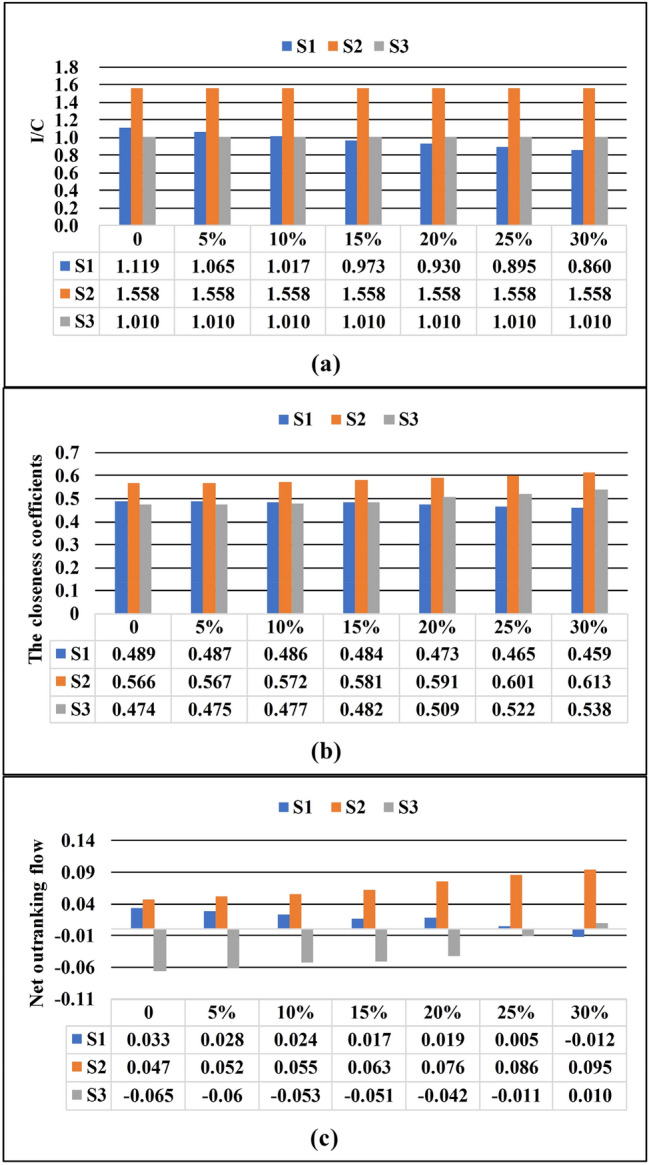
The final results for case 1 using the (**a**) CBA, (**b**) TOPSIS, and (**c**) PROMETHEE methods.

**Figure 7:**
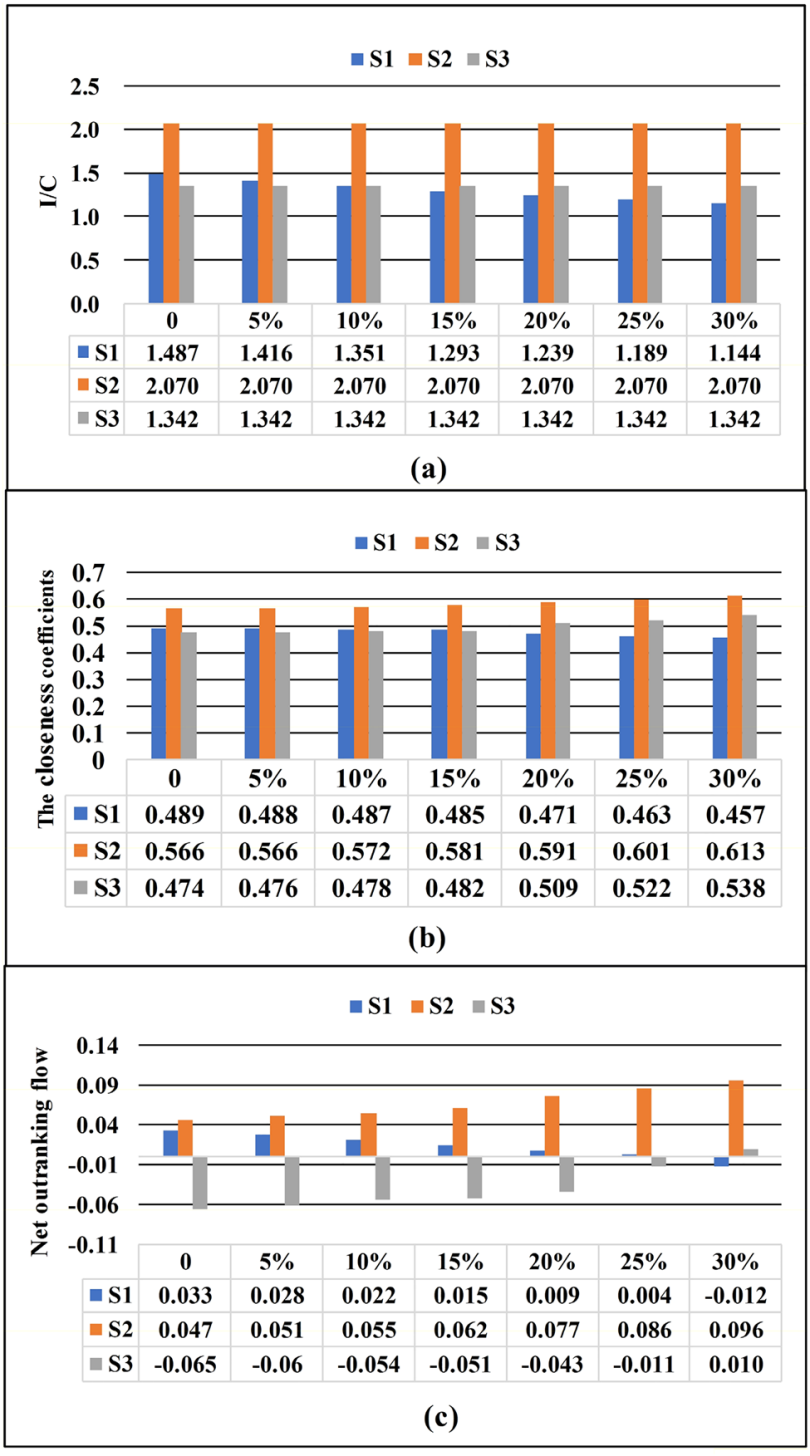
The final results for case 2 using the (**a**) CBA, (**b**) TOPSIS, (**c**) PROMETHEE methods.

In addition, the CBA method is more sensitive to cost changes than the traditional MCDM methods. To facilitate comparison, the same cost was determined for all site alternatives as a baseline to analyze the changes in the CBA, TOPSIS, and PROMETHEE results (Table 6). This paper presents two cases: Case 1 keeps the initial cost of the three site alternatives at the minimum cost ($2.98 million), and then scales up the cost of S1. To ensure the stability of the results, case 2 keeps the initial cost of the three site alternatives at the maximum cost ($3.96 million), and then scales up the cost of S1. Figures 6 and 7 show the results of the CBA, TOPSIS, and PROMETHEE methods for cases 1 and 2, respectively. Obviously, for the CBA method, the ranking results of the three site alternatives changed when the cost of S1 is increased by 10–15% for both cases 1 and 2.

**Table 6:** The final ranking of the TOPSIS, PROMETHEE, and CBA methods (when all costs are the minimum or maximum cost of the site alternative).

Site alternatives	TOPSIS		PROMETHEE		CBA method			
	Closeness coefficients	Ranking	Net outranking flow	Ranking	IofAs	Cost (million dollar)	I/C (IofAs divided by Cost)	Ranking
**Case 1**					**Minimum cost**			
S1	0.489	2	0.033	2	4.43	2.98	1.119	2
S2	0.566	1	0.047	1	6.17	2.98	1.558	1
S3	0.474	3	− 0.065	3	4.00	2.98	1.010	3
**Case 2**					**Maximum cost**			
S1	0.489	2	0.033	2	4.43	3.96	1.487	2
S2	0.566	1	0.047	1	6.17	3.96	2.070	1
S3	0.474	3	− 0.065	3	4.00	3.96	1.342	3

For the TOPSIS and PROMETHEE methods, when the results changed, the cost of S1 increases ranged from 15 to 20% and 25 to 30% respectively. Moreover, the results remain the same when the initial cost base of the site alternative increases (case 2). This indicates that CBA method makes the result more sensitive to the change in cost. The main reason is that the advantages of S1 in other aspects make up for S1's disadvantages in cost to varying degrees in the TOPSIS and PROMETHEE methods. Therefore, S1 will not be considered the worst option unless its cost increases so dramatically that the cost disadvantage outweighs the other advantages. For the CBA method, cost is an independent parameter and will not be interfered by other factors, which fully reveals the impact of the cost on the results. As a result, when evaluating projects with high cost, the results of the CBA method will enable decision-makers to fully consider the cost factor to reduce project risks.

### Sensitivity analysis

To ensure the reliability of the CBA results and to reduce the influence of decision-makers' subjectivity on the results, five scenarios are designed to investigate how the results fluctuate when one factor's advantages change in this study. We altered the relative advantages of the social, environmental, economic, technological, and geographical factors. The details of the scenarios are as follows:

- Scenarios A: Modify the IofAs of the factors associated with the social factors proportionately while maintaining the IofAs of the other factors.
- Scenarios B: Modify the IofAs of the factors associated with the environmental factors proportionately while maintaining the IofAs of the other factors.
- Scenarios C: Modify the IofAs of the factors associated with the economic factors proportionately while maintaining the IofAs of the other factors.
- Scenarios D: Modify the IofAs of the factors associated with the technological factors proportionately while maintaining the IofAs of the other factors.
- Scenarios E: Modify the IofAs of the factors associated with the geographical factors proportionately while maintaining the IofAs of the other factors.

**Figure 8:**
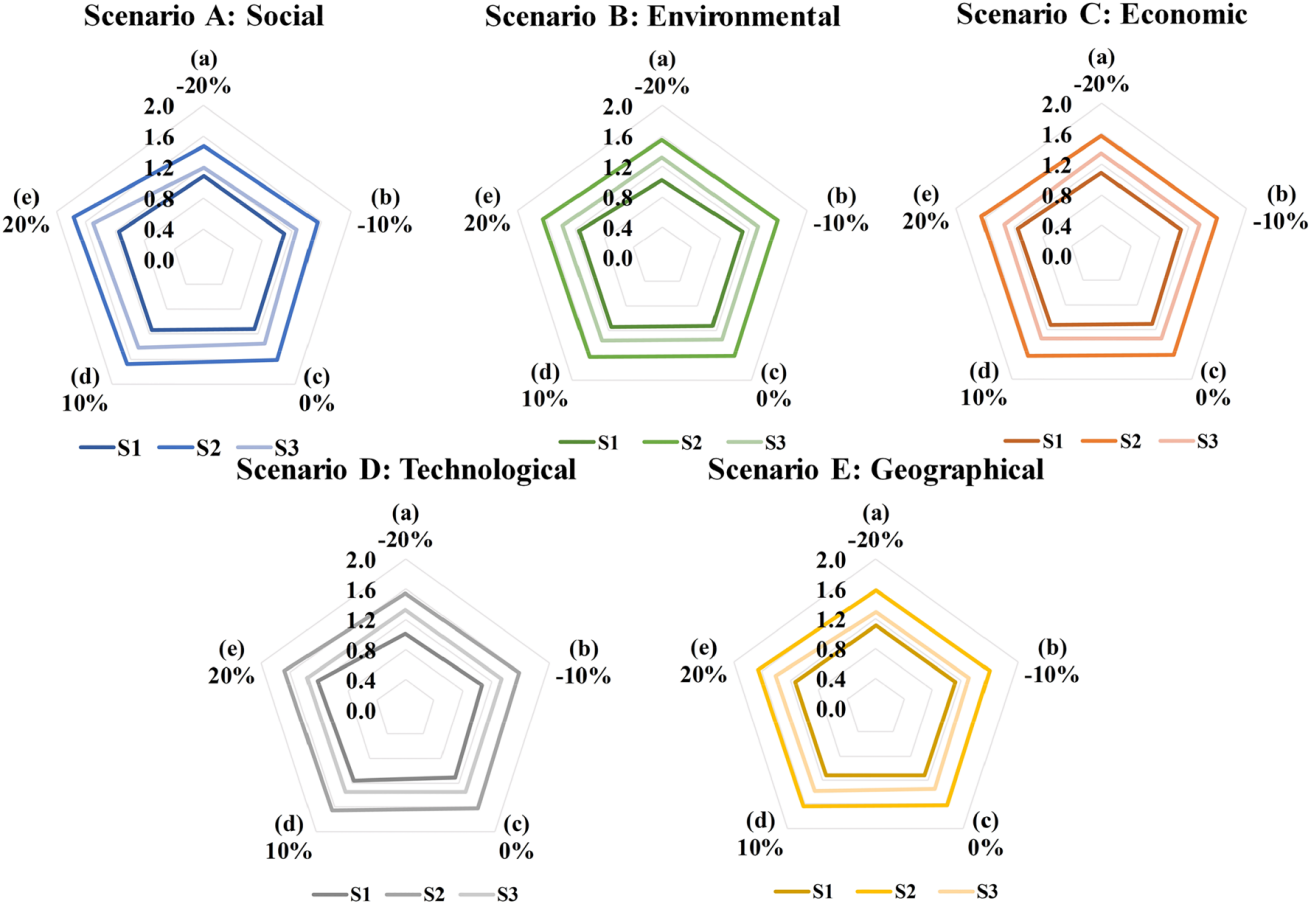
The sensitivity analysis results for scenarios A to E.

Tables 7 and 8 show the IofAs and I/C values for each site alternative in the five different scenarios. The resulting rankings for the five scenarios are displayed in Fig. 8, which illustrates that when the IofAs of the five factors were changed, the CBA results were all S2 > S1 > S3, indicating that the CBA results were stable in this study. Moreover, S1 and S3 are sensitive to social and technological factors. When the value of IofAs for social factors decreases or the value of IofAs for technological factors increases, the values of I/C for S1 and S3 get progressively closer.

**Table 7:** The values of IofAs for the different scenarios.

	(a) − 20%	(b) − 10%	(c) 0%	(d) + 10%	(e) + 20%
**Scenario A (social)**					
S1 (IofAs)	4.290	4.360	4.430	4.500	4.570
S2 (IofAs)	5.620	5.895	6.170	6.445	6.720
S3 (IofAs)	3.530	3.765	4.000	4.235	4.470
**Scenario B (environmental)**					
S1 (IofAs)	4.010	4.365	4.430	4.495	4.560
S2 (IofAs)	5.900	6.108	6.170	6.232	6.294
S3 (IofAs)	3.940	3.955	4.000	4.045	4.090
**Scenario C (economic)**					
S1 (IofAs)	4.274	4.352	4.430	4.508	4.586
S2 (IofAs)	6.010	6.090	6.170	6.250	6.330
S3 (IofAs)	4.000	4.000	4.000	4.000	4.000
**Scenario D (technological)**					
S1 (IofAs)	4.010	4.220	4.430	4.640	4.850
S2 (IofAs)	5.900	6.035	6.170	6.305	6.440
S3 (IofAs)	3.940	3.970	4.000	4.030	4.060
**Scenario E (geographical)**					
S1 (IofAs)	4.390	4.410	4.430	4.450	4.470
S2 (IofAs)	6.040	6.105	6.170	6.235	6.300
S3 (IofAs)	3.820	3.910	4.000	4.090	4.180

**Table 8:** The values of I/C and the final ranking results for the different scenarios.

	(a) − 20%	(b) − 10%	(c) 0%	(d) + 10%	(e) + 20%	Rank					
							(a)	(b)	(c)	(d)	(e)
**Scenario A: social**											
S1 (I/C)	1.083	1.101	1.119	1.136	1.154	S1	3	3	3	3	3
S2 (I/C)	1.471	1.543	1.615	1.687	1.759	S2	1	1	1	1	1
S3 (I/C)	1.185	1.263	1.342	1.421	1.500	S3	2	2	2	2	2
**Scenario B: environmental**											
S1 (I/C)	1.013	1.102	1.119	1.135	1.152	S1	3	3	3	3	3
S2 (I/C)	1.545	1.599	1.615	1.631	1.648	S2	1	1	1	1	1
S3 (I/C)	1.322	1.327	1.342	1.357	1.372	S3	2	2	2	2	2
**Scenario C: economic**											
S1 (I/C)	1.079	1.099	1.119	1.138	1.158	S1	3	3	3	3	3
S2 (I/C)	1.573	1.594	1.615	1.636	1.657	S2	1	1	1	1	1
S3 (I/C)	1.342	1.342	1.342	1.342	1.342	S3	2	2	2	2	2
**Scenario D: technological**											
S1 (I/C)	1.013	1.066	1.119	1.172	1.225	S1	3	3	3	3	3
S2 (I/C)	1.545	1.580	1.615	1.651	1.686	S2	1	1	1	1	1
S3 (I/C)	1.322	1.332	1.342	1.352	1.362	S3	2	2	2	2	2
**Scenario E: geographical**											
S1 (I/C)	1.109	1.114	1.119	1.124	1.129	S1	3	3	3	3	3
S2 (I/C)	1.581	1.598	1.615	1.632	1.649	S2	1	1	1	1	1
S3 (I/C)	1.282	1.312	1.342	1.372	1.403	S3	2	2	2	2	2

## Conclusion

This paper begins with the discussion of CE and considers that choosing an optimal site for solar power plants is an important way to promote the CE of renewable energy. Considering the high cost for the construction of solar power plants, this paper separates the cost from the other factors in the process of solar power plant site selection to provide investors with the maximum investment return. Considering the complexity of solar power plant construction, this study proposes a scheme that incorporates the CBA method and a solar power plant model involving economic, technological, geographical, environmental, and social factors to provide technical support for optimal site selection in California. This scheme also provides a new way of thinking for investors to realize the CE of solar energy.

This study has also demonstrated that the CBA method has a good performance in the decision-making of the optimal site selection for solar power plants. In the scenarios set up in this article, the appropriate ranking for the site alternatives using the CBA method is S2 > S1 > S3. The results show that the CBA method can provide more transparent and objective decision-making than the traditional MCDM methods, making the task easier for the decision-makers. The CBA method can fully reflect the impact of cost on decision-making and allows the experts and stakeholders to make a sagacious choice based on the cost analysis to reduce the risk of the project.

The main limitation of this article is that it does not discuss the treatment of PV modules after the end of the lifespan of a solar power plant. Future research will closely link the optimal site selection of solar power plants by considering waste recycling and other relevant factors related to the CE.

## Data Availability

The data used in the publication were made from meteorology and geography. It is widely mentioned in the “Methodology” section of the article.
